# A Composite of Cubic Calcium-Magnesium Sulfate and Bioglass for Bone Repair

**DOI:** 10.3389/fbioe.2022.898951

**Published:** 2022-06-07

**Authors:** Yan Chen, Tie Zhang, Qi Zhang, QingJian Lei, ShiJie Gao, KangWen Xiao, FeiFei Yan, Lin Cai

**Affiliations:** ^1^ Department of Spine Surgery and Musculoskeletal Tumour, Zhongnan Hospital of Wuhan University, Wuhan, China; ^2^ Hubei Osteolink Biomaterial Co, Ltd. (Wuhan Hi-tech Research Center of Medical Tissues), Wuhan, China

**Keywords:** calcium sulfate, bioglass, magnesium, osteogenesis, bone reparation

## Abstract

Calcium sulfate (CS) bone cement has been shown to have good biocompatibility and can be used as a bone filler for repairing bone defects. However, its clinical application is limited due to its low compressive strength and weak bone repair activity. To this end, in this study, cubic crystalline magnesium-doped calcium sulfate (MgCS) was prepared and mixed with 45S5 bioglass (BG) to form a composite bone cement (MgCS/BG). The results show that cubic crystal calcium sulfate helps to increase the compressive strength of the composite bone cement to more than 60 MPa. More importantly, the obtained magnesium-doped composite bone cement can promote the adhesion and differentiation of mesenchymal stem cells and has good bioactivity. Through a skull defect model, it was found that MgCS/BG can significantly enhance bone defect repair and new bone formation. This new composite MgCS/BG is very promising for future translation into clinical applications.

## 1 Introduction

Calcium sulfate (CS) bone cement has received extensive attention from researchers due to its nontoxic properties and high biocompatibility (Nilsson et al., 2013). At present, CS has been widely used as a material for dental impression and orthopaedic casting (Zhang et al., 2015; Subramaniam et al., 2016). However, CS is not widely used as a substitute for bone grafting (Qi et al., 2013; Li et al., 2014). This is mainly due to the low mechanical strength of CS, which is not stable at the implant site (Hu et al., 2012). Moreover, CS is not recommended for the treatment of major bone defects due to its lack of biological activity, which is not conducive to early bone regeneration *in vivo* (Kim et al., 2018; Nabiyouni et al., 2018). In recent years, there has been renewed interest in CS, mainly because the coagulation mechanism of CS does not involve acid-base reactions and does not affect other biologically active substances (Chen et al., 2018; Lin et al., 2021). Therefore, CS can be used as a potential carrier of various proteins or drugs.

To realize the application of CS as a bone graft, it is necessary to improve both the mechanical and biological properties. Magnesium ions are vital for bone health, and approximately 60% of the magnesium ions in the body are stored in the bone matrix (de Baaij et al., 2015). Magnesium deficiency can lead to bone dysplasia, poor mechanical strength of bone, and loss of bone mineral density (Zhang et al., 2019). Previous studies have shown that the addition of magnesium ions in the preparation of calcium sulfate can change its crystal form, converting it to cubic crystal form (Nieves, 2014; Yan et al., 2021). Cubic calcium sulfate particles can effectively decrease the porosity and specific surface area, which help to increase the mechanical properties of bone cement (Parvan et al., 2021). Therefore, the construction of cubic Mg-doped calcium sulfate is of great significance for promoting the application of bone cement in bone transplantation and bone repair.

As calcium sulfate has no biological activity, does not contain phosphate ions and degrades quickly, it is still difficult to have good osteogenic ability under the condition of high strength. Bioglass is a promising bone repair material with high bioactivity and a suitable rate of degradation (Hsu et al., 2019). However, because it cannot be self-cured, it cannot properly fill and repair bone defects of different shapes (Fiume et al., 2018). Therefore, combining it with injectable materials is an appropriate way to make it more clinically applicable.

In this study, we developed an improved CS formulation with the aim of enhancing the mechanical and biological properties of CS. The new formula is based on pure CS, adding magnesium ions to synthesize uniform cubic crystals at high temperature and high pressure (130 ± 2°C, 0.19 ± 0.01 MPa). To make the material biologically active and help cells adhere, 45S5 bioglass powder was mixed to obtain MgCS/BG. We expect that MgCS/BG can be used as a novel bone graft and bone repair material.

## 2 Materials and Methods

### 2.1 Preparation of MgCS and MgCS/BG

At room temperature, premixed sodium citrate (2 ± 0.05 g) and magnesium sulfate heptahydrate (25 ± 0.5 g) were added to pure water and stirred until the solution was completely clear. Then, calcium sulfate dihydrate powder (500 ± 2 g) was added and stirred for 20 min to obtain the reaction solution. Under high temperature and high pressure (130 ± 2°C, 0.19 ± 0.01 MPa), the reaction solution was allowed to react by itself for 5 h. After the reaction was completed, the supernatant was poured out to obtain a massive hemihydrate calcium sulfate precipitate, which was washed three times with boiling water, dried at 105°C for 4 h and crushed to obtain hemihydrate calcium sulfate powder, termed MgCS. The phase structure of MgCS powder was analysed and characterised by a D8 Advance X-ray diffraction (XRD) analyser (Bruker AXS)with the following analysis conditions: target Cu-Ka ray (2 = 0.15406 nm), tube pressure 40 kV, tube flow 40 mA, 20 scanning range 10°–80°. Finally, we mixed MgCS (80%) and BG powder (20%) with a ball-mill instrument to obtain MgCS/BG. All chemicals were purchased from Solarbio (BeiJing, China).

### 2.2 Scanning Electron Microscopy

A Zeiss Ultra Plus field emission scanning electron microscope (FE-SEM, magnification: 5000) was used to observe the microstructure of the MgCS and MgCS/BG bone cements. Energy dispersive spectrometry (EDS) was used to analyse the elemental composition of the test samples.

### 2.3 Injectability

The injectable property of the composite cement was tested. The cement homogenate was evenly mixed with 1 g of powder and approximately 300 µl of pure water. Then, the cement homogenate was put into a 1 ml syringe, and the air in the syringe was discharged to obtain 1 ml mud. After approximately 3 min, the cement was extruded, and the volume that could not be extruded was recorded as H. Then, the calculation formula of injectable (I) is I = (1-H) *100%.

### 2.4 Disintegration Resistance

The newly prepared cement was injected into normal saline at 37°C to test the cohesion or disintegration resistance of the cement in liquid. After soaking in physiological saline solution for 1 and 24 h and then placed on a shaker for half an hour, the state of bone cement in physiological saline solution was recorded with a camera. The turbidity of the physiological saline solution, sample integrity and disintegrated debris of the two groups were observed and compared. There was no cracking or disintegration of cements observed during the soaking.

### 2.5 The Curing Time

According to the GB/T-1346 standard, the curing time of bone cement was measured with a Vica needle. When the needle fell freely on the cement surface without obvious indentation, the final curing time was recorded. Each group had 5 replicates. The results were recorded as the mean ± SD.

### 2.6 Mechanical Properties

The strength of MgCS and MgCS/BG cement after curing at a loading rate of 0.5 mm/min was tested on a universal testing machine (Zoone Technology Co., LTD., China). The sample was prepared with a silica gel mould (diameter: 6 mm × 12 mm), and the experiment was repeated 5 times.

### 2.7 Degradation

To test the degradation behaviour of bone cement, a cylindrical cement sample (5 mm × 1 cm in diameter) was immersed in a physiological salt solution at 37°C (the ratio of solution volume to sample mass was 1 g/200 ml). Samples were removed on Days 1, 7, 14, 21 and 28 and dried at 60°C for 24 h. Five samples in each group were measured per immersion time, and the results were expressed as the mean ± standard deviation.

### 2.8 Biomineralization

For characterization of *in vitro* bioactivity, the 24 h-set cement was soaked in simulated body fluid (SBF) in a 37.0°C water bath for 14 days with a surface area-to-volume ratio of 0.1 cm^−1^. The surface morphology and chemical structures of MgCS/BG after SBF immersion were measured using scanning electron microscopy (SEM, HITACHI, S4800), and the elemental composition of the surface was analysed using EDS.

### 2.9 Haemocompatibility

To verify the blood compatibility of the bone cement, fresh blood from SD rats was used in the experiment. Ten millilitres of fresh blood was collected and diluted 1:1 with normal saline after heparin anticoagulation. The MgCS, BG and MgCS/BG extracts were each placed into 5 ml centrifuge tubes, as were 5 ml of physiological saline as a negative control and 5 ml of distilled water was used as a positive control. After each tube was preheated at 37°C for 30 min, 0.1 ml of freshly diluted anticoagulant blood was added to each tube, which was mixed and incubated in a water bath for 60 min. Finally, a small amount of solution was taken from each tube and smeared on a glass slide for observation under the microscope. The remaining solution was centrifuged at 2500 r/min for 5 min to observe the haemolysis of each tube. Three parallel experiments were conducted for each sample.

### 2.10 Cell Viability

Rat primary mesenchymal stem cells (BMSCs) were extracted for cell experiments. The sterilized MgCS and MgCS/BG samples were immersed in MEM Alpha medium at a rate of 0.2 g/ml according to ISO/EN 10993–5 and incubated at 37°C for 24 h to obtain the improved MEM Alpha medium. A total of 3.0 × 10^3^ cells were cultured in each well of 96-well plates for 24 h, and then 200 µl modified MEM α medium, 15% foetal bovine serum (Gibco, United States), 1% penicillin (Gibco, United States) and 1% streptomycin (Gibco, United States) were added. After incubation at 37°C for 1, 3 or 5 days with 95% air humidity and 5% carbon dioxide, the viability of the cells was determined by a cell count kit-8 (CCK-8) test, and the respective optical densities were measured at 450 nm using an enzyme immunoassay analyser and spectrophotometer. All experiments were performed in triplicate.

### 2.11 Cell Adhesion

BMSCs (1.0 × 10^5^) were inoculated on the surface of bone cement samples (circular wafer 6 mm in diameter) and cultured for 3 days. The bone cement sample was then soaked in 4% paraformaldehyde for 20 min, washed with PBS 3 times for 5 min each, and stained with FITC (Sigma–Aldrich, United States) for 20 min. The samples were washed with PBS 3 times and stained with DAPI for 5 min. Then, the samples were observed by confocal scanning microscopy. Finally, we counted the number of cells according to the number of blue cell nuclei using ImageJ software.

### 2.12 Osteogenic Differentiation

BMSCs were cultured in modified MEM Alpha medium for 3 days and then replaced with osteogenic differentiation medium (ScienCell Research Laboratories, United States) for 7 days for alkaline phosphatase (ALP) staining and 14 days for alizarin red (AR) staining. The cells were washed with PBS and fixed with 4% paraformaldehyde at 37°C for 15 min. The cells were stained with an alkaline phosphatase staining kit (Beyotime, China) and stained with 1% AR at room temperature for 15 min. The stained specimens were then observed with an inverted microscope (Olympus Corp, United States) and a digital camera.

### 2.13 Rat Skull Defect Model and Histopathological Staining

Eighteen SD rats (200–250 g) were used as critical skull defect models. After the rats were anaesthetized with IsoFlo (Isoflane, Abbott Laboratories, United Kingdom), a 6 mm full-thickness defect was drilled in the centre of the skull using a diamond drill and cooled by rinsing with saline. The rats were randomly divided into three groups based on different fillings. The defects of the blank group were not filled after surgery, and the MgCS group and MgCS/BG group each were filled with the corresponding preformed bone cements. At 6 and 12 weeks, three rats in each group were sacrificed, and the skull was completely removed. The skull tissues were examined by micro-CT and histological analysis (H&E and Masson staining). A software analysis system was used to quantitatively analyse the percentage of new bone in the total bone defect area.

### 2.14 Statistical Analysis

Two-way analysis of variance (ANOVA) was used for statistical analysis. All quantitative data are expressed as the mean ± SD. *p* < 0.05 was considered statistically significant.

## 3 Results

### 3.1 Characterization of Hexagonal MgCS

**FIGURE 1:**
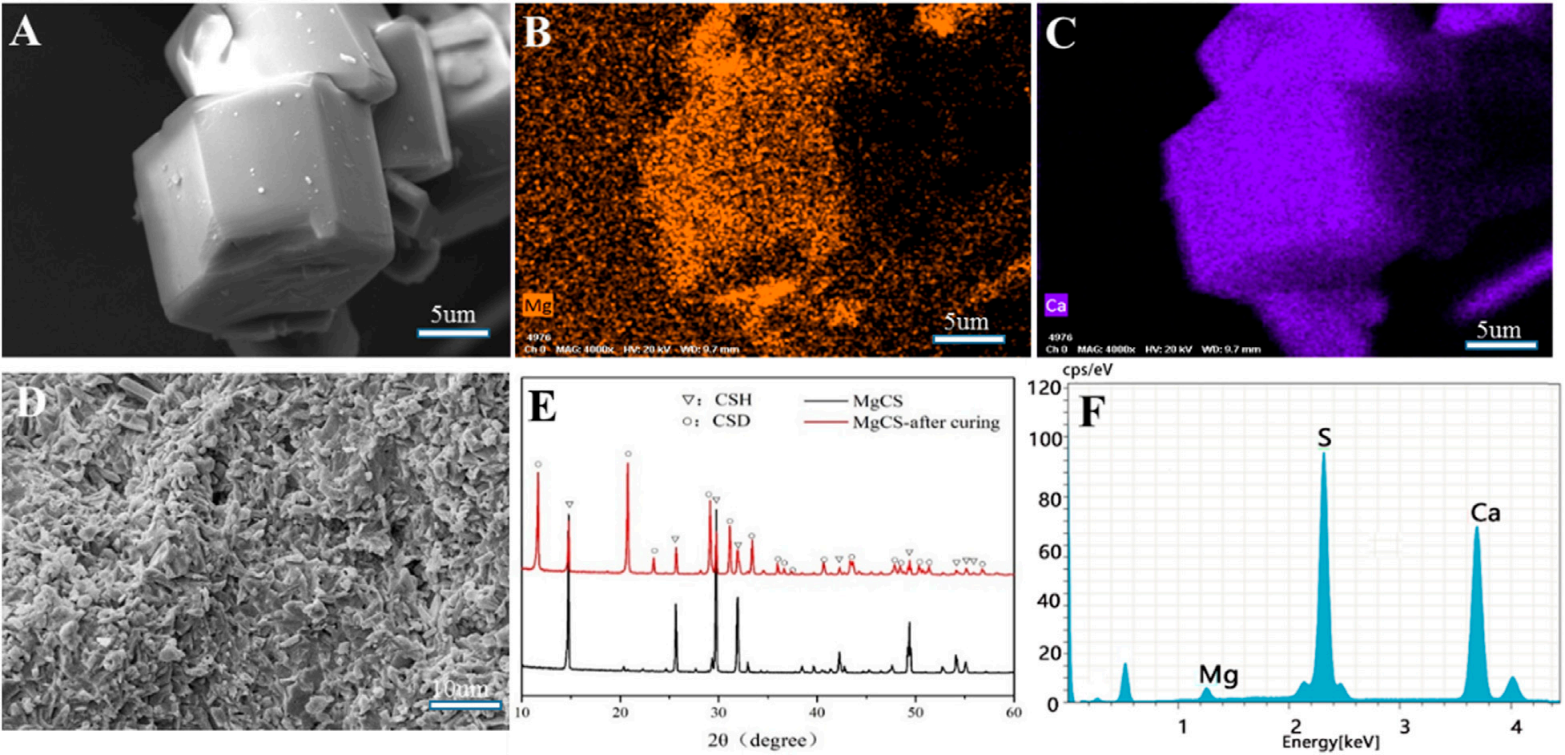
Characterization of MgCS. **(A)** SEM of hexagonal MgCS; **(B)** Element mapping (Mg) of hexagonal MgCS; **(C)** Element mapping (Ca) of hexagonal MgCS; **(D)** SEM of MgCS after curing; **(E)** XRD of MgCS before and after curing; **(F)** EDS spectra of hexagonal MgCS.

The SEM image shows that our magnesium-doped calcium sulfate (MgCS) is indeed a hexagonal crystal with a base length of 5 µm and a height of 15 µm (Figure 1A). Through element mapping analysis, it was determined that calcium sulfate is the main component in hexagonal MgCS, and calcium and magnesium are uniformly dispersed in the crystal form (Figures 1B,C). The EDS spectra of MgCS also confirm its elemental composition (Figure 1E). The SEM image of MgCS after curing is shown in Figure 1D. The XRD chromatogram showed a clear characteristic peak of α-hemihydrate calcium sulfate (Figure 1F), and the cured MgCS showed a mixture of calcium dihydrate and hemihydrate calcium sulfate, indicating that hemihydrate calcium sulfate was not completely transformed into calcium sulfate dihydrate after curing.

**FIGURE 2:**
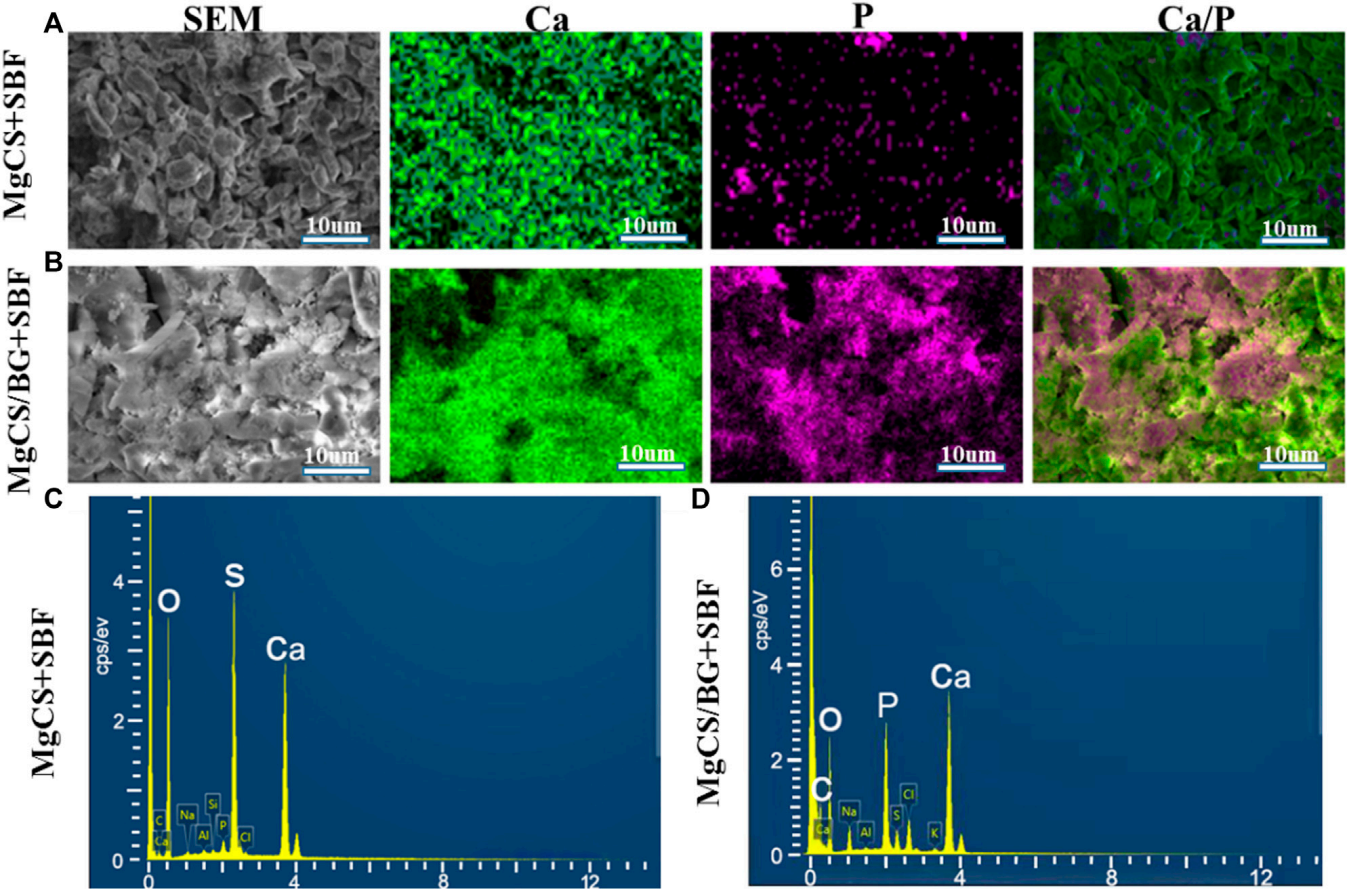
*In vitro* SBF study of cement. SEM and element mapping images of the surface of MgCS after 14 days of SBF **(A)**; SE SEM and element mapping images of MgCS/BG after 14 days of SBF **(B)**; EDS spectra of MgCS **(C)** and MgCS/BG **(D)** after 14 days of SBF incubation.

Next, we mixed hexagonal MgCS with bioglass to obtain a novel cement (MgCS/BG) and studied its biocompatibility. We first used simulated body fluids (SBF) to mineralize MgCS and MgCS/BG. After immersion in SBF for 14 days, a porous hydroxyapatite mineralized layer can be seen on the surface of MgCS/BG, but almost no such layer can be seen on the surface of MgCS (Figures 2A,B), and obvious phosphorus can be seen on the surface of MgCS/BG (Figures 2C,D). This shows that the mixing of hexagonal MgCS with bioglass is more helpful to simulate the mineralization of body fluids on its surface. Good mineralization properties are very important for promoting osteogenic differentiation *in vivo*.

**FIGURE 3:**
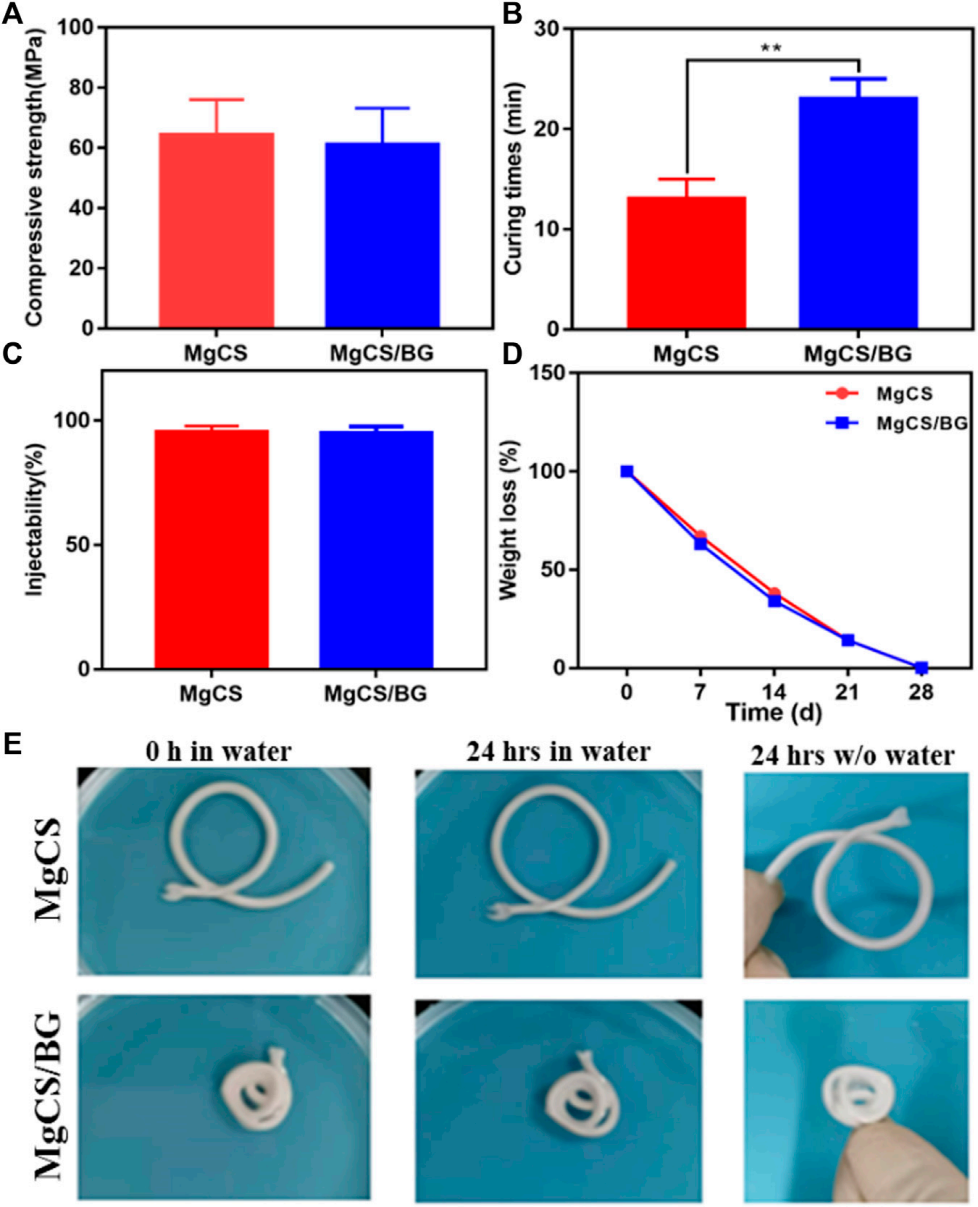
Physical properties of hexagonal MgCS and MgCS/BG. **(A)** Compressive strength of MgCS and MgCS/BG; **(B)** Curing times of MgCS and MgCS/BG; **(C)** Injectability of MgCS and MgCS/BG; **(D)** Weight loss of MgCS and MgCS/BG. **(E)** Morphology of hexagonal MgCS and MgCS/BG after immersion in water for 24 h.

We then further investigated the mechanical strength of MgCS and MgCS/BG. The results showed that the load-bearing capacity and mechanical strength of MgCS and MgCS/BG reached more than 60 MPa (Figure 3A), indicating that hexagonal MgCS has great potential to increase mechanical strength.

After that, we investigated the curing time of MgCS and MgCS/BG. As shown in Figure 3B, the curing time of MgCS is usually 14 min, and the curing time of MgCS/BG is approximately 25 min, which are both within a more suitable range. Generally, curing times that are too short or too long are not suitable for grafting operations. In terms of injectability, both MgCS and MgCS/BG reached 100% (Figure 3C).

To test the degradation of bone cement *in vitro*, we immersed MgCS/BG in physiological saline solution and measured its weight at different time points. After immersion in physiological saline solution for 24 h, the quality and surface morphology of MgCS and MgCS/BG did not change significantly (Figures 3D,E), indicating that both hexagonal MgCS and MgCS/BG have good disintegration resistance. After immersion in physiological saline solution for 7 days, MgCS/BG lost approximately 40% of its mass and completely disappeared after 28 days (Figure 3D).

### 3.2 *In Vitro* Bioactivity and Cell Biocompatibility

**FIGURE 4:**
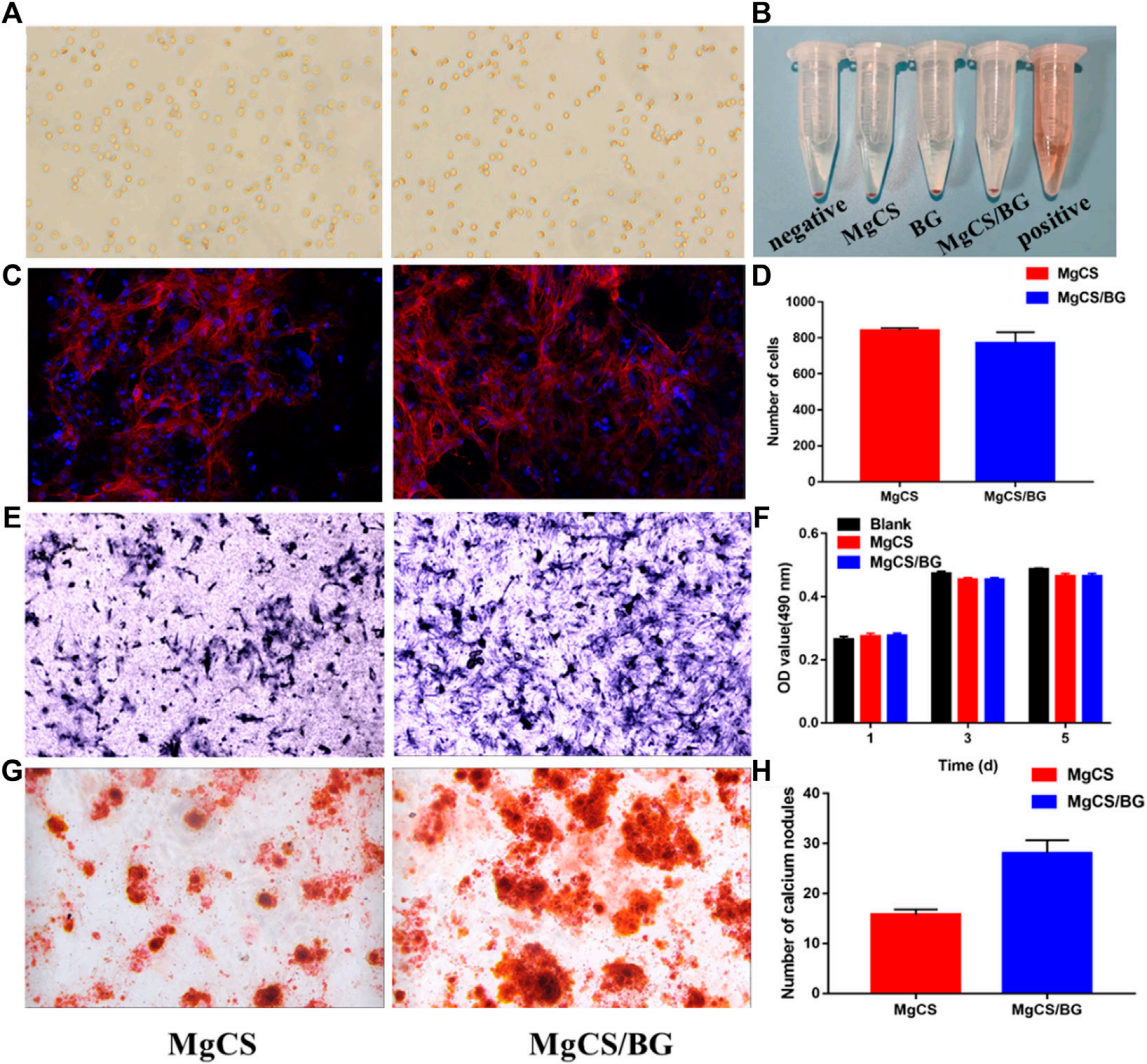
Bioactivity and cell biocompatibility of hexagonal MgCS and MgCS/BG. **(A)** Morphology of Red blood cells on MgCS and MgCS/BG; **(B)** Hemolysis of red blood cells on different groups; **(C)** Cell adhesions on MgCS and MgCS/BG; **(D)** Number of cells attached on MgCS and MgCS/BG; **(E)** Alkaline phosphatase staining, blue representing alkaline phosphatase; **(F)** CCK-8 assay on Day 1, 3 and 5 after incubation. **(G)** Alizarin red S staining, calcium nodules are stained red in extracellular matrix mineralization. **(H)** Number of calcium nodules in alizarin red staining.

Figure 4A shows the influence of MgCS and MgCS/BG extracts on the morphology of red blood cells. It can be seen that all red blood cells have the normal fovea disk shape. Figure 4B shows that the various components of cement do not cause erythrocyte lysis.

To further investigate the biocompatibility of MgCS/BG, we investigated cell adhesion on its surface. First, we used confocal microscopy to observe the adhesion of BMSCs on hexagonal MgCS and MgCS/BG (Figure 4C). Analysis of the number of cells by ImageJ software showed (Figure 4D) that the number of cells was similar in the two groups, but the pseudopodia in the MgCS/BG group had wider cell extension and better cell morphology. In the cytotoxicity experiments, we soaked MgCS/BG in cell culture medium and then investigated its effect on the proliferation of BMSCs. The results showed that MgCS/BG had no effect on cell proliferation (Figure 4E). These results indicate that MgCS/BG has high compatibility and low toxicity.

To verify the bioactivity of MgCS/BG, we also performed a staining assay for osteocyte-secreted proteins. Bone alkaline phosphatase is one of the phenotypic markers of osteoblasts and can directly reflect the activity and function of osteoblasts. We incubated MgCS/BG with osteocytes for 7 days, and the results showed that MgCS/BG could promote the secretion and expression of alkaline phosphatase (Figure 4F).

Calcium deposition is one of the hallmark events of cell maturation. To this end, we used alizarin red staining to study intracellular calcium deposition. The results showed that MgCS/BG was able to induce massive calcium deposition (Figures 4G,H).

### 3.3 *In vivo* Osteogenesis

**FIGURE 5:**
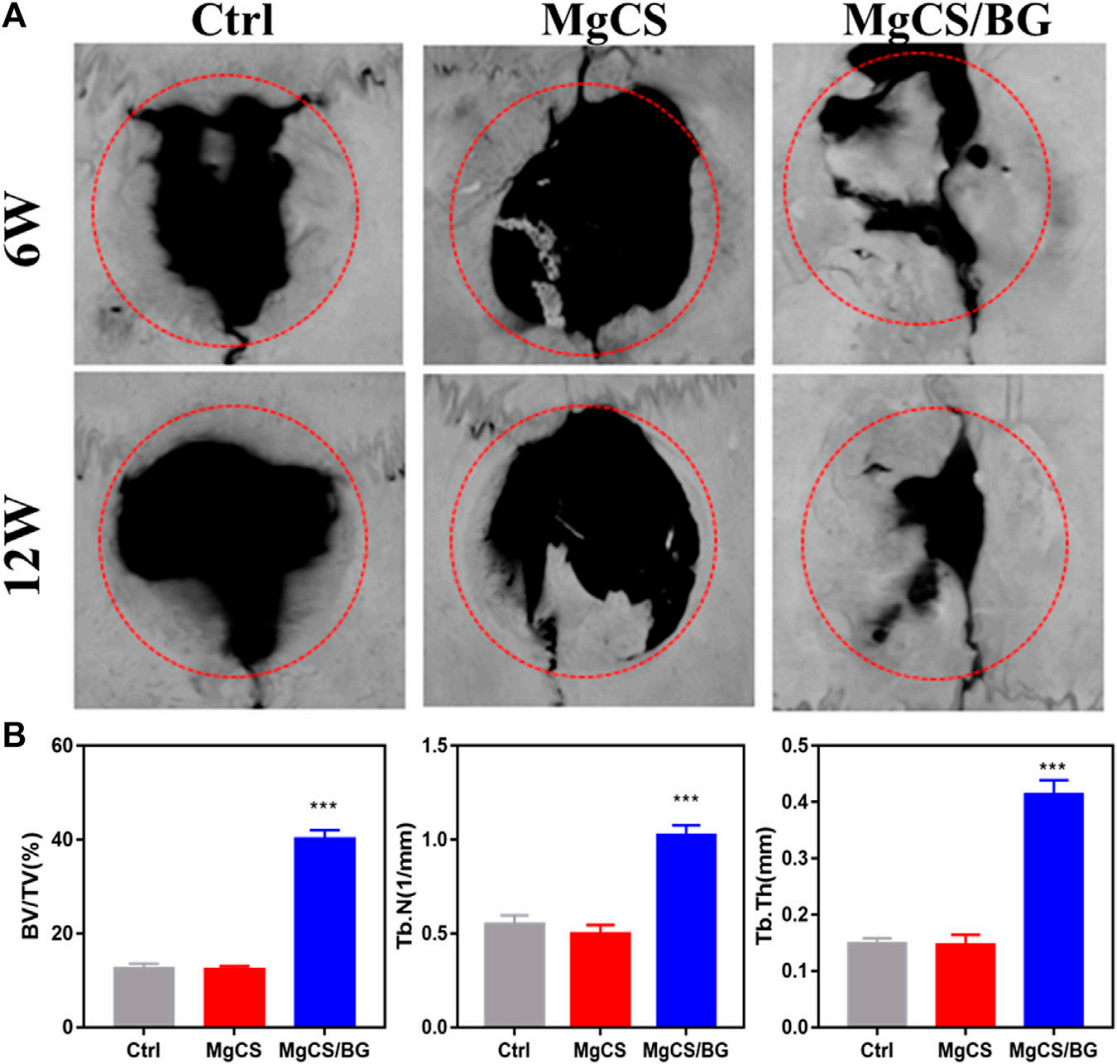
Effects of hexagonal MgCS and MgCS/BG on the skull defect model. **(A)** MicroCT after treatment for 6 or 12 weeks; **(B)** Quantitative analysis of new bone.

We studied the osteogenic properties of MgCS and MgCS/BG through a rat skull model. Figure 5 shows the micro-CT results of the different groups at 6 and 12 weeks. From the blank group and MgCS group, obvious bone defects were observed in both groups at 6 and 12 weeks, indicating that the 6 mm bone defect was critical and MgCS did not have good osteogenesis ability. By comparing MgCS and MgCS/BG, MgCS/BG showed obvious osteogenesis at 6 weeks and basically healed at 12 weeks. This was further confirmed by quantitative analysis. At 12 weeks, the BV/TV of MgCS/BG (39.7 ± 1.6%) was significantly higher than that of the MgCS group (12 ± 0.2%, *p* < 0.001). This indicates that MgCS alone does not have the effect of bone repair *in vivo*, and compared with the MgCS group, MgCS/BG has a better ability to promote bone regeneration.

**FIGURE 6:**
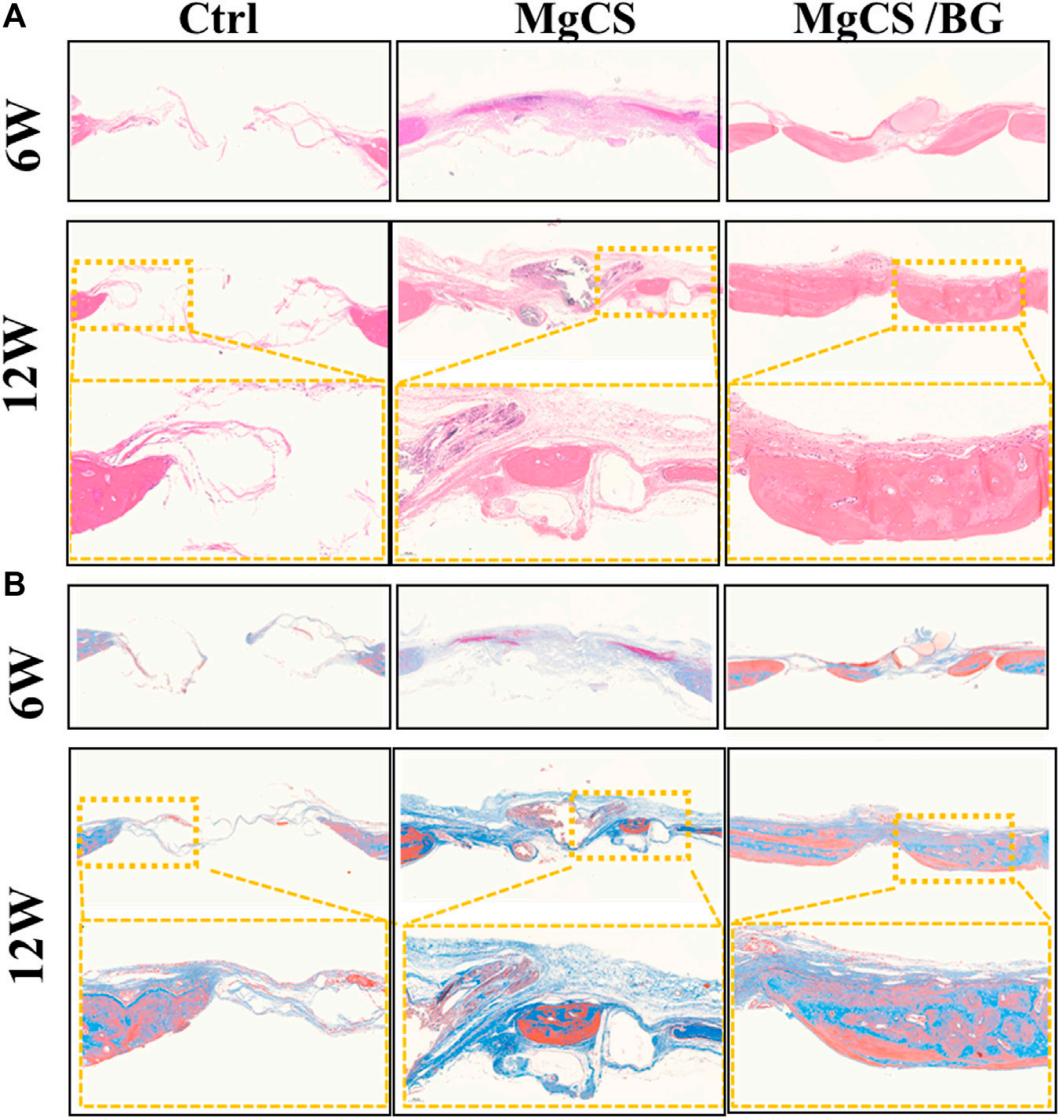
Histological analysis of the skull defect model after treatment with hexagonal MgCS and MgCS/BG. **(A)** HE staining; **(B)** Masson staining.

We also performed histological analysis by H&E and Masson staining at 6 and 12 weeks (Figure 6,). In the blank group, the bone defect was clearly demarcated, no obvious bone tissue growth was observed, and little neovascularization was observed at the bone defect. In the MgCS group, the material was basically degraded, a large amount of neovascularization could be seen at the bone defect, and a small amount of new bone tissue could be seen at 6 and 12 weeks. In the MgCS/BG group, a small amount of material was not degraded at 6 w, and a large amount of new bone tissue was observed. At 12 w, the material was basically degraded, and the new bone tissue basically covered the whole bone defect area. The results of H&E and Masson staining were consistent with the micro-CT observations.

**SCHEME 1:**
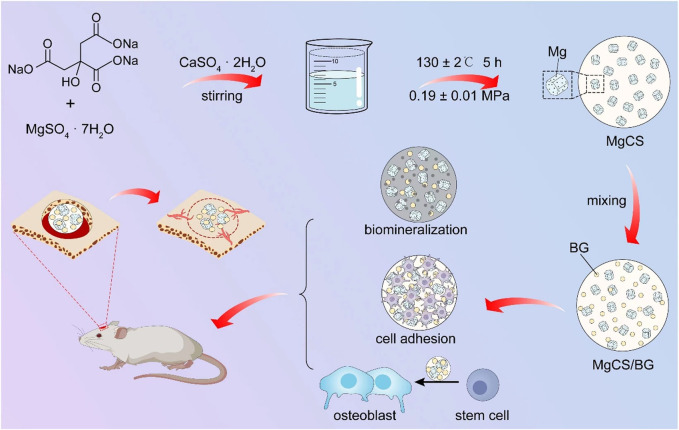
Schematic llustration for the Preparation of MgCS and MgCS/BG Bone Cement, and the Bone Formation Mechanism in the Presence of MgCS/BG.

## 4 Discussion

The clinical application of CSH is limited because of its weak strength, fast curing, rapid dissolution, and lack of biological activity (Cavelier et al., 2020; Xu et al., 2020). However, calcium sulfate can be applied in clinical practice mainly because its degradation rate and curing time can be delayed by optimizing its crystal phase, and better mechanical properties can be obtained. At the same time, due to the self-curing ability, by adding other biomaterials, CSH can achieve broader clinical application and better therapeutic outcomes (Yan et al., 2019). As showed in Scheme 1, the MgCS synthesized in this paper has short columnar hexagonal crystals. In our synthesis process, we added sodium citrate to change the growth rate of each crystal plane of calcium sulfate and magnesium sulfate to promote the formation of the hexagonal column structure and improve its crystallinity (Yazdimamaghani et al., 2017). In addition, the material was doped with magnesium to improve its osteogenic ability, mainly because magnesium can promote the differentiation of osteoblasts. The quantity of magnesium is second only to that of calcium and phosphorus in bones and is an essential element for the structure and function of bone cells (Holmes, 2016). Numerous studies have shown that magnesium promotes bone formation and regeneration and plays an important role in maintaining the strength and density of bones and teeth (Grünewald et al., 2016). XRD results show that MgCS is not completely transformed into calcium sulfate dihydrate after curing, which is mainly due to the dense crystal structure and small specific surface area of MgCS, which makes it difficult to react with water inside the crystal. This is also the reason why the curing time of MgCS is longer than that of traditional hemihydrate calcium sulfate. SEM results also show that the crystals are densely packed after solidification, which is also the reason for its good mechanical strength.

Clinically, many bone graft materials are a combination of various materials, which can achieve better osteogenic effects by combining the advantages of each material (Gurumurthy et al., 2020). Calcium sulfate alone is weakly acidic and has no biological activity (Xu et al., 2019). Therefore, adverse reactions and poor osteogenic effects of calcium sulfate materials can be seen clinically. Bioglass is alkaline, bioactive and degrades at a moderate rate; at the same time, it can form strong bonds with bone and soft tissue *in vivo* (Hu et al., 2021). Thus, by incorporating bioglass, it can increase the biological activity of the material and also neutralize the pH, delaying the degradation rate so that it has better biocompatibility and osteogenic ability. For these reasons, MgCS/BG did not inhibit cell growth but promoted the differentiation of mesenchymal stem cells and the expression of alkaline phosphatase. As shown in Figure 4, MgCS/BG has good biological activity, mainly because calcium sulfate can quickly release calcium ions to compensate for the slow release of calcium ions in bioglass, and the two together improve the biological activity of the material.

Due to the rapid degradation rate of calcium sulfate, it can generally be completely degraded within 2 months, and the degradation rate is faster than the rate of osteogenesis, resulting in poor bone conduction ability (Hao et al., 2018). This is also the reason why the MgCS group failed to achieve good osteogenesis. The addition of bioglass delays the degradation rate of the material, giving the material a certain bone conduction ability and biological activity and, furthermore, the surface of the porous hydroxyapatite structure is conducive to cell adhesion. The release of Mg ions can also promote mesenchymal stem cell differentiation to endow MgCS/BG with good osteogenic potential. The reason MgCS/BG and MgCS degrade at the same rate *in vitro* is that BG will separate from calcium sulfate when it degrades. *In vivo*, however, it can be observed that the degradation rate of MgCS/BG is significantly lower than that of MgCS, and the rate of degradation of MgCS/BG is basically the same as the rate of osteogenesis.

## 4 Conclusions

In conclusion, we changed the crystal shape of calcium sulfate by adding magnesium ions, thus obtaining a new calcium sulfate with mechanical strength much higher than ordinary calcium sulfate. After combination with bioglass, the new calcium sulfate composite cement not only has high strength but also promotes the adhesion and differentiation of mesenchymal stem cells. *In vitro* and *in vivo* experiments have verified its excellent potential for promoting bone repair. Our modification of bone cement has good potential for clinical translation, and it is believed that this research will soon be pushed into clinic applications.

## Data Availability

The raw data supporting the conclusions of this article will be made available by the authors, without undue reservation.
